# When to apply immune checkpoint inhibitor in patients with refractory advanced gastric cancer

**DOI:** 10.7150/jca.62853

**Published:** 2021-07-25

**Authors:** Hongsik Kim, Hana Kim, Minsang Lee, Minsuk Kwon, Jung Yong Hong, Jeeyun Lee, Ho Yeong Lim, Won Ki Kang, Seung Tae Kim

**Affiliations:** Division of Hematology-Oncology, Department of Medicine, Samsung Medical Center, Sungkyunkwan University School of Medicine, Seoul, Republic of Korea

**Keywords:** Gastric cancer, Immune checkpoint inhibitor, Nivolumab, Pembrolizumab, Biomarker

## Abstract

**Background:** Immune checkpoint inhibitors (ICIs) show clinical benefit in patients with refractory advanced gastric cancer (GC). The ICIs in routine clinical practice have been used in various treatment lines. Therefore, we investigated the timing for application of ICI in patients with refractory advanced GC.

**Methods:** We analyzed 187 patients with refractory advanced or recurrent GC who received ICIS as a 3rd- or 4th-line treatment between September 2015 and October 2020. Clinical outcomes of overall survival (OS), progression-free survival (PFS), objective response rate (ORR), and disease control rate (DCR) were evaluated.

**Results:** Among 187 patients, 105 received ICIs as a 3rd-line treatment and 82 as a 4th line. The ORR for ICIs was 10.5% (11/105) in 3rd line and 8.5% (7/82) in 4th line. The DCR for ICIs was 36.2% (38/105) in 3rd-line treatment and 31.7% (26/82) in 4th line. There was no significant difference for ORR (*P* = 0.819) or DCR (*P* = 0.870). The median PFS and OS to ICIs was 1.4 months (95% CI, 1.1 to 1.8 months) and 4.4 months (95% CI, 1.6 to 7.2 months) in 3rd line and 1.8 months (95% CI, 1.4 to 2.3 months) and 2.8 months (95% CI, 2.2 to 3.4 months) in 4th line. The median PFS and OS to ICIs was not different between 3rd line and 4th line (*P* = 0.495 and *P*=0.208, respectively). There were also no significant difference for PFS and OS between PD-L1-positive tumors (CPS≥1) and PD-L1-negative tumors (*P* = 0.910 and *P*=0.931, respectively).

**Conclusions:** ICIs showed similar clinical benefits in the 3rd-line and 4th-line settings. ICIs might be a reasonable approach for patients with refractory GC in the setting of 3rd-line or 4th-line treatment options.

## Introduction

Gastric cancer (GC) is the sixth most common cancer and the second leading cause of cancer death worldwide 1. In Korea, GC is the most incident cancer and the fourth leading cause of cancer death 2. In National Comprehensive Cancer Network (NCCN) and European Society for Medical Oncology (ESMO) guidelines, palliative chemotherapy is a standard care in patients with metastatic GC. The standard first-line chemotherapy is the combination of platinum and pyrimidine analogues, and common second lines are a combination of paclitaxel and ramucirumab, paclitaxel, docetaxel or irinotecan 3, 4. Despite these treatments, most patients with advanced GC experience disease progression, and prognosis is very poor.

Recently, immune checkpoint inhibitors (ICIs) showed clinical benefit in patients with refractory advanced GC 5. The ATTRACTION-2 (ONO-4538-12) study showed clinical benefit of nivolumab, a human IgG4 monoclonal antibody inhibitor of programmed death-1 (PD-1), in GC patients who had failed two or more treatments. The objective response rate (ORR) of nivolumab was 11.2 %, and median overall survival (OS) showed improvement compared to placebo (5.3 versus 4.1 months), regardless of positivity for programmed death-ligand 1 (PD-L1) 6. Also, the KEYNOTE-059 study reported the clinical benefit of pembrolizumab, another human IgG4 monoclonal antibody inhibitor of PD-1, in refractory GC patients as salvage therapy. The ORR of pembrolizumab was 11.6 % in all enrolled patients and 15.5 % in patients with PD-L1-positive tumors 7.

However, the optimal timing of the application of ICIs has not been determined, and numerous clinical trials are on-going. In clinical practice, the ICIs in routine clinical practice have been used in various treatment lines.

Herein, we investigated the timing of application of ICI in patients with refractory advanced GC.

## Patients and methods

### Patients

From September 2015 to October 2020 at Samsung Seoul Medical Center, 187 refractory GC patients treated with ICIs after progression on 2nd- or 3rd-line therapy were analyzed in this study. The following clinicopathologic characteristics were collected for all 187 patients: age, sex, number of metastatic sites, microsatellite instability (MSI), Epstein-Barr virus (EBV) in situ, positivity for PD-L1, and information on chemotherapy. The study protocol was approved (#2021-01-053) by the Institutional Review Board of Samsung Medical Center (Seoul, Korea), and was conducted in accordance with the ethical principles of the Declaration of Helsinki and the Korea Good Clinical Practice guidelines.

### Chemotherapy

Patients included in this study received nivolumab or pembrolizumab as ICI according to the physician. Nivolumab was administered at a dose of 3mg/kg intravenously every 2 weeks or pembrolizumab at a dose of 200mg intravenously every 3 weeks.

### Statistics

Descriptive statistics were used to summarize patient and tumor characteristics. Categorical variables were analyzed by chi-square test. Survival analyses were performed using the Kaplan-Meier method, and differences were analyzed by log-rank test. Hazard ratios and corresponding 95% confidence intervals were calculated using the Cox proportional hazards model. Progression-free survival (PFS) was defined as the time from the start of ICI until the date of disease progression or death from any cause. Univariate analysis was performed using Cox proportional hazards models for PFS and OS. Significant prognostic variables in univariate analysis for survival were included in the multivariate analysis. All *P*-values were two-sided, and statistical significance was set at *P* < 0.05. Statistical analysis was performed using IBM SPSS statistics version 25.

## Results

### Patient characteristics

Between September 2015 and October 2020, we analyzed 187 patients with refractory advanced or recurrent GC with immune checkpoint inhibitor (ICI), of whom 105 received ICI as a 3rd line and 82 received ICI as a 4th line treatment. Patient characteristics are presented in **Table 1**. Of patients with ICI as 3rd line, the median age was 57 years (range of 27 to 82 years), and 89 (84.8%) patients had two or more distant metastatic sites. Of patients with ICI as 4th line, the median age was 57 years (range of 29 to 78 years), and 80 (97.6%) patients had two or more distant metastatic sites. Status of MSI-H and EBV positivity were not different in patients with ICIs of 3rd or 4th line. However, positivity of PD-L1 expression was significantly higher in patients with ICI as 3rd line than in those with ICI as 4th line.

### Treatment outcomes with ICIs between 3rd- and 4th-line treatment

The median duration of follow-up was 3.0 months (IQR 1.4 to 7.5 months) in patients with ICI as 3rd-line treatment and 2.7 months (IQR 1.2 to 5.6 months) in those with 4th line. The overall response rate (ORR) for ICIs was 10.5% (11/105) in 3rd line and 8.5% (7/82) in 4th line (*P* = 0.819). The DCR for ICIs was 36.2% (38/105) in 3rd line and 31.7% (26/82) in 4th line (*P* = 0.870). Between 3rd and 4th lines, there was no difference in efficacy of ICIs (**Table 2**).

**Figure 1:**
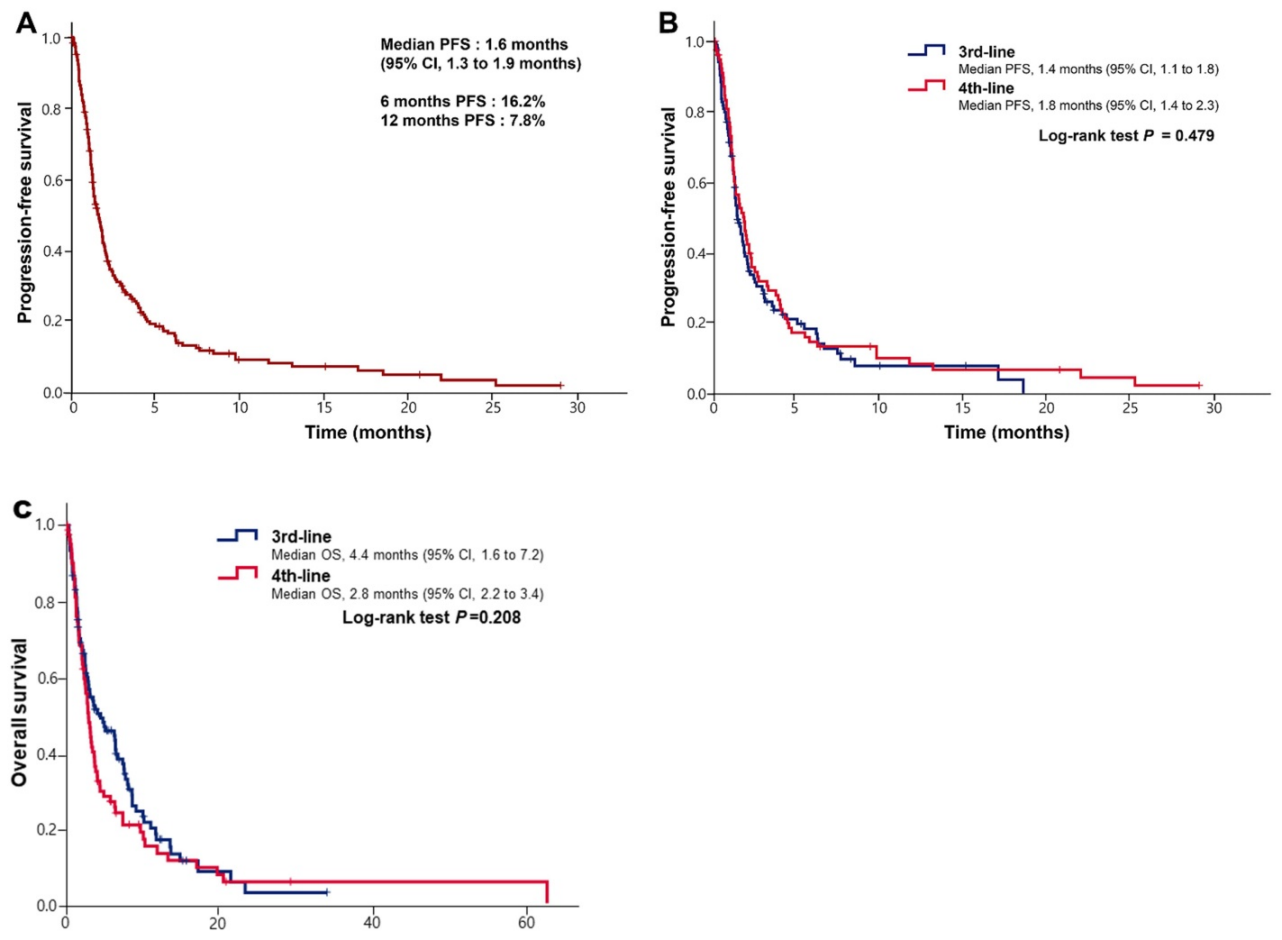
(A) Kaplan-Meier curves of progression-free survival (PFS) after immune checkpoint inhibitors. (B) Kaplan-Meier curves of progression-free survival (PFS) after immune checkpoint inhibitors according to lines of treatment. (C) Kaplan-Meier curves of overall survival (OS) after immune checkpoint inhibitors according to lines of treatment**.**

The median PFS after ICI was 1.6 months (95% CI, 1.3 to 1.9 months) (**Figure 1A**). The median PFS was 1.4 months (95% CI, 1.1 to 1.8 months) in 3rd-line treatment and 1.8 months (95% CI, 1.4 to 2.3 months) in 4th-line treatment, with no statistical difference (*P* = 0.495) (**Figure 1B**). The median PFS in patients with PD-L1-positive tumors (CPS≥1) was 2.0 months (95% CI, 1.1 to 3.0 months), while that in negative tumors was 1.9 months (95% CI, 1.3 to 2.5 months) (*P* = 0.910). The median OS was 4.4 months (95% CI, 1.6 to 7.2 months) in 3rd-line treatment and 2.8 months (95% CI, 2.2 to 3.4 months) in 4th-line treatment, with no statistical difference (*P*=0.208, **Figure 1C**). We found that 18 of 187 patients (9.6%) achieved the long term survival of more than 1 year. Furthermore, 7 of 187 patients (3.7%) had received ICIs for more than 1 year, and 3 continued to do so to present. Of 18 patients with long-term survival, 2 of 12 patients (16.7%) were EBV positivity, 4 of 9 patients (44.4%) were PD-L1 expression and 0 of 7 (0%) patients were MSI-H.

### Analysis of prognostic factors for PFS and OS after the starting ICIs

We conducted the univariate analysis for PFS and OS after starting ICIs to identify significant prognostic factors (**Table 3, Table 4**). In univariate analysis, age (≥ 65 vs. < 65) was only significant prognostic factor to PFS (*P*=0.003). Age (≥ 65 vs. < 65) and No. of metastatic sites (<2 vs. ≤2) were significantly associated with OS (*P*=0.018 and *P*=0.012, respectively). The timing of starting ICIs as 3rd or 4th line was not associated with PFS and OS, in univariate analysis. Multivariate analysis showed that age (*P* = 0.030) and No. of metastatic sites (*P* = 0.019) were significant independent prognostic factors for OS.

## Discussion

The ICIs have been used in various treatment lines in clinical practice for patients with AGC. Herein, we analyzed 187 refractory advanced or recurrent GC patients with ICI, of whom 105 received ICI as a 3rd line and 82 received ICI as a 4th line treatment. The ORR for ICIs was 10.5% (11/105) in 3rd line and 8.5% (7/82) in 4th line. The median PFS and OS to ICIs was not different between 3rd line and 4th line (*P* = 0.495 and *P*=0.208, respectively). These finding suggested that ICI could be one of reasonable options at both 3rd line and 4th line therapies in refractory AGC patients.

Previous studies have shown the efficacy and safety of ICIs as 2nd-, 3rd- or later line treatment in refractory GC patients, consistent with the present studies. For example, in the ATTRACTION-2 study, the median OS and PFS were 5.3 months and 1.6 months, respectively, and the ORR to nivolumab was 11% 6. In the KEYNOTE-059 study, the median OS and PFS were 5.6 months and 2.0 months, respectively, and the ORR to pembrolizumab was 12% 7. Systematic review and meta-analysis of ICIs reported that anti PD-1 inhibitor improved the long-term clinical benefit. However, ICIs were used as various treatment lines in previous studies 5. Previous clinical trials showed subgroup analysis for the optimal timing of the application of ICIs. In the ATTRACTION-2 study, subgroup analysis for OS showed that patients with nivolumab as 4th-line treatment had better survival than patients with nivolumab in the 2nd or 3rd line (Hazard ratio 0.44 versus 0.82 and 0.89, respectively) 6, and the ORR of pembrolizumab was better in the setting of 3rd line than in 4th or higher (ORR 16.4% versus 6.4%) in the KEYNOTE-059 study 7. The timing of the application of ICIs was different between two clinical trials. Based on our real world data, ICIs might be a reasonable option in both the 3rd line and 4th line for refractory GC patients.

Previous research has reported that ICIs were more effective in patients with PD-L1 positivity, MSI-H, or EBV positivity 5, 8, 9. In the present study, 80 patients were assessed for expression of PD-L1 using CPS. There was no difference in PFS to ICIs according to the status of PD-L1 expression and between patients with EBV-positive and -negative tumors (*P* = 0.509). This discrepancy might be caused by heterogeneous patients' characteristics and small sample size.

In the present study, although ICIs were used as 3rd- or 4th-line therapy, 18 of 187 patients (9.6%) achieved long-term survival of greater than 1 year. Furthermore, 7 of 187 patients (3.7%) received ICIs for more than 1 year, and 3 continued to do so to present. This finding is concordant with previous studies reporting a long-term clinical benefit of ICI 6, 10, 11. Considering that patients received ICIs as 3rd or 4th line therapy, these findings were very interested. Further, we must conducted the prospective biomarker research to select AGC patients who achieve the long term survival.

This analysis has limitations. First, it was a retrospective nature with a clinically heterogeneous population that is subject to potential biases. Second, the study included a relatively small number of patients, making it difficult to draw definite conclusions. Third, only Asian patients with GC were analyzed in the study, limiting generalizability because of differences in molecular profiles and clinical features between Western and Eastern patients with GC. Therefore, our findings must be interpreted with caution.

In conclusion, ICIs showed similar clinical outcomes between 3rd-line and 4th-line settings. ICIs might be a reasonable approach for refractory AGC patients in such settings.

## Author Contributions

Conception and design: Hongsik Kim and Seung Tae Kim, Provision of study materials or patients: Hongsik Kim, Hana Kim, Minsang Lee, Minsuk Kwon, Jung Yong Hong, Jeeyun Lee, Ho Yeong Lim, Won Ki Kang and Seung Tae Kim, Collection and assembly of data: Hongsik Kim, Data analysis and interpretation: Hongsik Kim and Seung Tae Kim, Manuscript writing: All authors, Final approval of manuscript: All authors.

## Figures and Tables

**Table 1:** Patient characteristics

Patient characteristics	3rd line (n = 105)	4th line (n = 82)	*P*-value
**Median age (years)**	57 (27 - 82)	57 (29 - 78)	0.349
**Patients aged <65 years**	76 (72.4%)	64 (78.0%)	0.375
**Sex**			0.209
Male	57 (54.3%)	52 (63.4%)	
Female	48 (45.7%)	30 (36.6%)	
**Immune checkpoint inhibitor**			0.156
Nivolumab	72 (68.6%)	48 (58.5%)	
Pembrolizumab	33 (31.4%)	34 (41.5%)	
**Organs with metastases**			0.003
<2	16 (15.2%)	2 (2.4%)	
≥2	89 (84.8%)	80 (97.6%)	
**Previous therapies**			
Any	105 (100%)	82 (100%)	
Pyrimidine analogues	105 (100%)	82 (100%)	
Platinum	102 (97.1%)	80 (97.6%)	
Taxane	90 (85.7%)	81 (98.8%)	
Irinotecan	14 (13.3%)	68 (82.9%)	
Ramucirumab	76 (72.4%)	60 (73.2%)	
**Microsatellite instability**			0.381
MSS	84 (80.0%)	64 (78.0%)	
MSI-H	2 (1.9%)	0 (0%)	
Not evaluable	19 (18.1%)	18 (22.0%)	
**EBV in situ**			0.621
No	69 (65.7%)	49 (59.8%)	
Yes	4 (3.8%)	5 (6.1%)	
Not evaluable	32 (30.5%)	28 (34.1%)	
**PD-L1 22C3 IHC CPS**			0.062
PD-L1<1	21 (20.0%)	16 (19.5%)	
1≤PD-L1<10	24 (22.9%)	5 (6.1%)	
10≤PD-L1	8 (7.6%)	6 (7.3%)	
Not evaluable	52 (49.5%)	55 (67.1%)	

*MSS* Microsatellite stable, *MSI-H* Microsatellite instability-high, *EBV* Epstein-Barr virus, *PD-L1* Programmed death-ligand 1, *IHC* Immunohistochemistry, *CPS* Combined positive score.

**Table 2:** Objective response rate and survival data

	3^rd^ line (n = 105)	4^th^ line (n = 82)	*P*-value
Complete response	0	0	
Partial response	11 (10.5%)	7 (8.5%)	
Stable disease	27 (25.7%)	19 (23.2%)	
Progressive disease	47 (44.8%)	34 (41.5%)	
Not evaluable	20 (19.0%)	22 (26.8%)	
Objective response rate	11 (10.5%)	7 (8.5%)	0.819
Disease control rate	38 (36.2%)	26 (31.7)	0.870

**Table 3:** Univariate analysis of progression-free survival after ICIs

Variables	No.	Median PFS (months, 95% CI)	Univariate Analysis	
			HR (95% CI)	*P*-value
**Median age, years**				0.003
< 65	140	1.4 (1.1 - 1.7)	1	
≥ 65	47	2.6 (0.0 - 5.5)	0.57 (0.39 - 0.83)	
**Sex**				0.209
Male	109	1.5 (1.2 - 1.9)	1	
Female	78	1.8 (1.0 - 2.7)	0.82 (0.60 - 1.12)	
**Immune checkpoint inhibitors**				0.502
3^rd^ line	105	1.4 (1.1 - 1.8)	1	
4^th^ line	82	1.8 (1.4 - 2.3)	0.90 (0.66 - 1.23)	
**No. with metastatic sites**				0.086
<2	18	3.1 (0.7 - 5.4)	1	
≥2	169	1.5 (1.2 - 1.8)	1.60 (0.94 - 2.73)	
Microsatellite instability*				0.327
MSS	148	3.7 (2.6 - 4.7)	1	
MSI-H	2	1.2 (0.6 - 1.7)	2.02 (0.50 - 8.23)	
Not evaluable	37			
**EBV in situ**				0.509
No	118	1.7 (1.3 - 2.0)	1	
Yes	9	3.2 (0.0 - 6.4)	0.77 (0.36 - 1.67)	
Not evaluable	60			
**PD-L1 22C3 IHC CPS ≥ 1**				0.921
No	37	1.9 (1.3 - 2.5)	1	
Yes	43	2.0 (1.1 - 3.0)	0.97 (0.59 - 1.60)	
Not evaluable	107			
**PD-L1 22C3 IHC CPS ≥ 10**				0.957
No	66	1.9 (1.4 - 2.4)	1	
Yes	14	1.3 (0.1 - 2.4)	1.02 (0.54 - 1.92)	
Not evaluable	107			

*HR* Hazard ratio, *MSS* Microsatellite stable, *MSI-H* Microsatellite instability-high, *EBV* Epstein-Barr virus, *PD-L1* Programmed death-ligand 1, *IHC* Immunohistochemistry, *CPS* Combined positive score. * Subgroup analysis of microsatellite instability indicates the estimated mean PFS due to low incidence of MSI-H.

**Table 4:** Univariate and multivariate analysis of overall survival after ICIs

Variables	No.		Median OS (months, 95% CI)	Univariate Analysis		Multivariate Analysis		
				HR (95% CI)	*P*-value	HR (95% CI)	*P*-value	
**Median age, years**					0.018			0.030
< 65	140		2.7 (2.2 - 3.3)	1		1		
≥ 65	47		2.6 (0.0 - 5.5)	1.61 (1.08 - 2.38)		0.65 (0.44 - 0.96)		
**Sex**					0.265			
Male	109		3.1 (2.4 - 3.8)	1				
Female	78		3.1 (1.8 - 4.2)	0.82 (0.60 - 1.15)				
**Immune checkpoint inhibitors**					0.209			
3^rd^ line	105		4.4 (1.6 - 7.2)	1				
4^th^ line	82		2.8 (2.2 - 3.4)	1.23 (0.89 - 1.70)				
**No. of metastatic sites**					0.012			0.019
<2	18		8.5 (4.4 - 12.5)	1		1		
≥2	169		2.8 (2.4 - 3.3)	2.14 (1.18 - 3.88)		2.05 (1.13 - 3.71)		
Microsatellite instability*					0.962			
MSS	148		7.4 (4.8 - 10.1)	1				
MSI-H	2		4.8 (2.1 - 7.4)	1.04 (0.26 - 4.20)				
Not evaluable	37							
**EBV in situ**					0.465			
No	118		3.3 (2.6 - 4.1)	1				
Yes	9		6.2 (0.0 - 16.1)	0.75 (0.35 - 1.63)				
Not evaluable	60							
**PD-L1 22C3 IHC** **CPS ≥ 1**					0.931			
No	37		3.9 (2.3 - 5.6)	1				
Yes	43		3.3 (0.9 - 5.8)	1.02 (0.61 - 1.73)				
Not evaluable	107							
**PD-L1 22C3 IHC** **CPS ≥ 10**					0.917			
No	66		3.1 (2.5 - 3.8)	1				
Yes		14	3.0 (0.8 - 5.3)	1.04 (0.53 - 2.01)				
Not evaluable		107						
								

*HR* Hazard ratio, *MSS* Microsatellite stable, *MSI-H* Microsatellite instability-high, *EBV* Epstein-Barr virus, *PD-L1* Programmed death-ligand 1, *IHC* Immunohistochemistry, *CPS* Combined positive score. * Subgroup analysis of microsatellite instability indicates the estimated mean PFS due to low incidence of MSI-H

## Abbreviations

ICI: immune checkpoint inhibitors
GC: gastric cancer
OS: overall survival
PFS: progression-free survival
ORR: objective response rate
DCR: disease control rate
NCCN: National Comprehensive Cancer Network
ESMO: European Society for Medical Oncology
PD-1: programmed death-1
PD-L1: programmed death-ligand 1
MSI: microsatellite instability
EBV: Epstein-Barr virus
